# Courtship and spawning behaviour of medaka in a semi-outdoor environment initiating at midnight

**DOI:** 10.1038/s41598-025-01037-8

**Published:** 2025-05-16

**Authors:** Yuki Kondo, Satoshi Awata

**Affiliations:** Laboratory of Animal Sociology, Department of Biology, Graduate School of Science, Osaka Metropolitan University, Osaka, 558-8585 Japan

**Keywords:** Spawning, Courtship, Model organism, Medaka, *Oryzias latipes*, Video observation, Behavioural ecology, Freshwater ecology, Animal behaviour

## Abstract

Reproductive timing is a critical ecological trait that directly influences fitness. Medaka (*Oryzias latipes*), a small freshwater fish, is widely used as a model organism in various scientific fields. However, ecological studies conducted under (semi-) natural conditions remain limited. Although, spawning has been reported to occur within 1 h before and after sunrise, direct observations remain scarce. We investigated the timing of spawning initiation and associated courtship in medaka through 24-h observations using infrared cameras under semi-natural conditions. During the experiments, sunrise occurred at approximately 4:45. Observations of the 31 pairs revealed that spawning occurred between 1:05–9:48, with a peak at 2:00–4:00. Unlike previous reports but like the most recent fieldwork, only 26% of the total spawning events (8/31) were observed within 1 h before and after sunrise. Male courtship behaviours, including following females and quick circle displays, increased from midnight, peaking between 2:00–5:00. This study provides new insights into the natural reproductive timing of medaka, suggesting a possible adaptation to nocturnal spawning, likely as a strategy to reduce predation on both parents and eggs. It also underscores the importance of investigating the ecology of model organisms under (semi-)natural conditions to gain a more comprehensive understanding of biological phenomena observed in laboratory settings.

**Supplementary Information:**

The online version contains supplementary material available at 10.1038/s41598-025-01037-8.

## Introduction

Reproduction timing is a critical ecological trait that directly influences fitness. Various taxonomic groups exhibit time-specific reproductive behaviours, such as those specialised for the nocturnal and dawn periods Numerous examples across animal taxa illustrate the importance of reproductive timing, including the synchronised spawning of corals during the full moon at night^1,2^, nocturnal and dawn spawning in many fish species^3^, nocturnal group breeding behaviour in frogs^4,5^, and peak vocalisation activity at dawn in birds^6,7^. Nocturnal reproduction is thought to have evolved under multiple selective pressures such as predator avoidance, environmental adaptation, mate synchronisation, and optimal conditions for offspring survival^1,3^. In particular, predation pressures are suggested to be an important factor in the evolution of nocturnal spawning in many externally fertilising fishes, as the reduced activity of diurnal animals during the night enhances the survival rates of both eggs and parent fishes^8,9^. Helfman suggested that nocturnal spawning might be widespread among fish^3^. Still, systematic research on fish ecology at night remains limited because of the challenges of collecting observational data in darkness^10^.

Medaka (*Oryzias latipes*) is a small (~ 40 mm), externally fertilised fish established as a crucial model organism across diverse research fields, including physiology, genetics, developmental biology, behavioural science, and medicine. However, despite over a century of use as a model organism, much remains unclear regarding its ecology and behaviour in wild and semi-outdoor environments, particularly its reproductive ecology^11^. Medaka is distributed across Japan, inhabiting still- or slow-flowing waters, such as rice paddies, ponds, and irrigation channels^12^, with an approximate lifespan of one year in the wild^13^. The breeding season of medaka spans from early summer to autumn^14–17^. Under laboratory conditions, medaka spawning events were observed every morning, with males engaging in multiple spawning events and females spawning once daily^18,19^. Spawning typically occurs in pairs with the external fertilisation of eggs. The female carries the fertilised eggs on her abdomen and, after a few hours, brushes against aquatic plants to attach the eggs to the foliage. Owing to the ease of observing medaka spawning events, numerous behavioural and ecological studies have been conducted in the laboratory, including studies on female mate choice^20–24^, male mate guard behaviour^25–27^, alternative reproductive tactics for males^28–30^, and sperm allocation^31,32^. All studies were conducted under bright lighting conditions in the morning.

In previous laboratory studies, medaka spawning has been reported to occur within an hour before or after light onset^33–36^. Kobayashi et al. (2012)^37^ have been the only studies to describe spawning behaviours in the wild based on visual observations, indicating that spawning events occur mainly from early morning to mid-morning after sunrise. However, these studies did not directly observe spawning under dark conditions; instead, they estimated the spawning time based on post-spawning egg developmental stages or visually observed daytime spawning behaviours. Consequently, the timing of spawning initiation remains unclear. Laboratory studies have shown that females complete ovulation at night to prepare for spawning events in the morning^33^. Medaka activity levels significantly increased approximately 4–5 h before light onset, which could be associated with behaviours such as searching for mating partners^38^. Medaka can also spawn in the dark^39^ and engage in spawning events without relying on visual cues^40^.

Building on these laboratory findings, Kondo et al. (2025)^12^ conducted nocturnal field observations using video cameras and found evidence that medaka in a population initiated spawning earlier than previously thought, with higher activity levels, increased courtship behaviour, and females carrying fertilised eggs after spawning, all around midnight (to 1 h before sunrise). However, their study has two limitations^12^. First, spawning time was estimated indirectly by observing females with fertilised eggs rather than actual spawning behaviours. Second, their observations were limited to 21:00 to 5:00, preventing a comprehensive analysis of courtship dynamics over a 24-hour cycle. These issues must be overcome by determining the precise timing of spawning and the changes in the reproductive activity of this species.

This study aimed to elucidate the temporal dynamics of spawning initiation and associated courtship behaviours in medaka during the reproductive period. Through continuous 24-h observations under semi-natural conditions (natural water temperature and photoperiod resembling outdoor conditions during the breeding season), this study aimed to illuminate previously undocumented aspects of spawning and courtship behavioural patterns in medaka.

## Methods

### Maintenance

Medaka (himedaka) acquired from a local pet shop were maintained for one month under the following conditions: eight stock breeding tanks (90.5 × 60.5 × 21.0 cm, length × width × height) were set up outdoors on the campus of Gifu University, with approximately 100 individuals housed in each tank. The fish were maintained under natural photoperiod and ambient temperature conditions, with Tetra Kilimin (Tetra, Melle, Germany) administered three times daily as a dietary supplement. Spawning was observed almost every day in the stock breeding tanks.

### Experimental methods

Mating experiments were conducted during the peak of the medaka breeding season, from July 6 to 12 and July 17 to 20 2024, under shaded conditions in an outdoor area without streetlights on the Gifu University campus, utilising natural light and water temperatures. The experimental tanks used were 22.5 × 15.8 × 5.5 cm (length × width × height) and were filled with 950 mL of water. The water depth was 2.7 cm, which was suitable for observing spawning behaviour^31^.

A male and female, taken from separate breeding tanks, were introduced into the experimental tank between 17:00 and 18:00 h. Thirty-one pairs (1–5 per day) were created (Supplementary Table S1). A 24-h continuous recording was performed at 18:00 using an FDR-AX60 video recorder (Sony, Tokyo, Japan). From 18:00 to 6:00, infrared light (ICAMI, Tokyo, Japan) illuminated the tank, and video recordings were conducted in night mode. From 6:00 to 18:00, the light was turned off, and video recording was performed under natural light in normal mode. The infrared light (850 nm) was outside the visible spectrum of the medaka, thereby minimising behavioural effects^41–44^. No feed was provided to the fish during the experiments. The experiment ended at 18:00 the following day.

After the experiment, anaesthesia was administered following the manufacturer’s protocol using FA100 (DS Pharma Animal Health, Osaka, Japan) diluted 1:2000, and body mass was measured using an electronic balance HT-120 (A & D, Tokyo, Japan). After measurements, the individuals were returned to the holding tanks and were not reused in subsequent experiments.

## Analysis of spawning event and courtship behaviour

The video data were analysed using ELAN 6.8 annotation software. To determine the timing of spawning, we identified the time the medaka spawned from the video. A series of spawning behaviours, including egg and sperm release, were determined as described by Kondo et al. (2020)^32^.

Next, to examine temporal changes in behaviour over 24 h, a 10-min video clip was extracted from 10 to 30 min of each hour. Twenty-four video clips were collected for analysis for each male-female pair. Medaka spawning behaviour proceeds in a sequence of events^12,32,45,46^: the male pursues the female (following), the male swims rapidly around the female (quick circle), the male encircles the female with its dorsal and anal fins (wrapping), the female releases eggs, the male releases sperm (egg and sperm release), and the male separates from the female (leaving). After spawning, the female carried the eggs attached to her abdomen for several hours, rubbing her abdomen against the aquatic plants and depositing the eggs onto the plants. Based on previous studies^12,31,45,46^, two courtship behaviours were quantified within the 10-minute video clips: duration of following (where the male followed the female) and frequency of quick circles (where the male circled in front of the female).

### Data analysis

In this study, the mean male body mass was 0.30 g (0.05 SD, range = 0.21–0.40, *n* = 31), and the mean female body mass was 0.33 g (0.05 SD, range = 0.24–0.42, *n* = 31; Supplementary Table S1). Temperature data (average, minimum, and maximum) during the study period were obtained from the Japan Meteorological Agency website (https://www.data.jma.go.jp/obd/stats/etrn/view/nml_sfc_ym.php?prec_no=52&block_no=47632&year=&month=&day=&view=), and sunrise and sunset times were acquired from the National Astronomical Observatory of Japan website (https://eco.mtk.nao.ac.jp/koyomi/dni/2023/dni22.html*)* (Supplementary Table S2).

All statistical analyses were conducted using R 4.4.1 (R Core Team, 2024)^47^. Peak spawning events were determined by gamma distribution fitting. GAMM was implemented to examine temporal changes in courtship behaviour (following and quick circles) over 24 h. Model significance was evaluated using likelihood ratio tests, with the significance level set at *p* < 0.05. First, we examined the temporal variation in the total duration of the following behaviour within the 10-min video segments. The response variable was the total duration, and the explanatory variable was each time point. A GAMM assuming a Gaussian distribution was developed with male ID included as a random effect. Second, we analysed temporal changes in the frequency of courtship displays within 10-minute video segments. The response variable was the frequency of courtship displays, and the explanatory variable was each time point. Assuming a negative binomial distribution, a GAMM was constructed with male ID as a random effect.

## Results

### Timing of spawning events

**Fig. 1:**
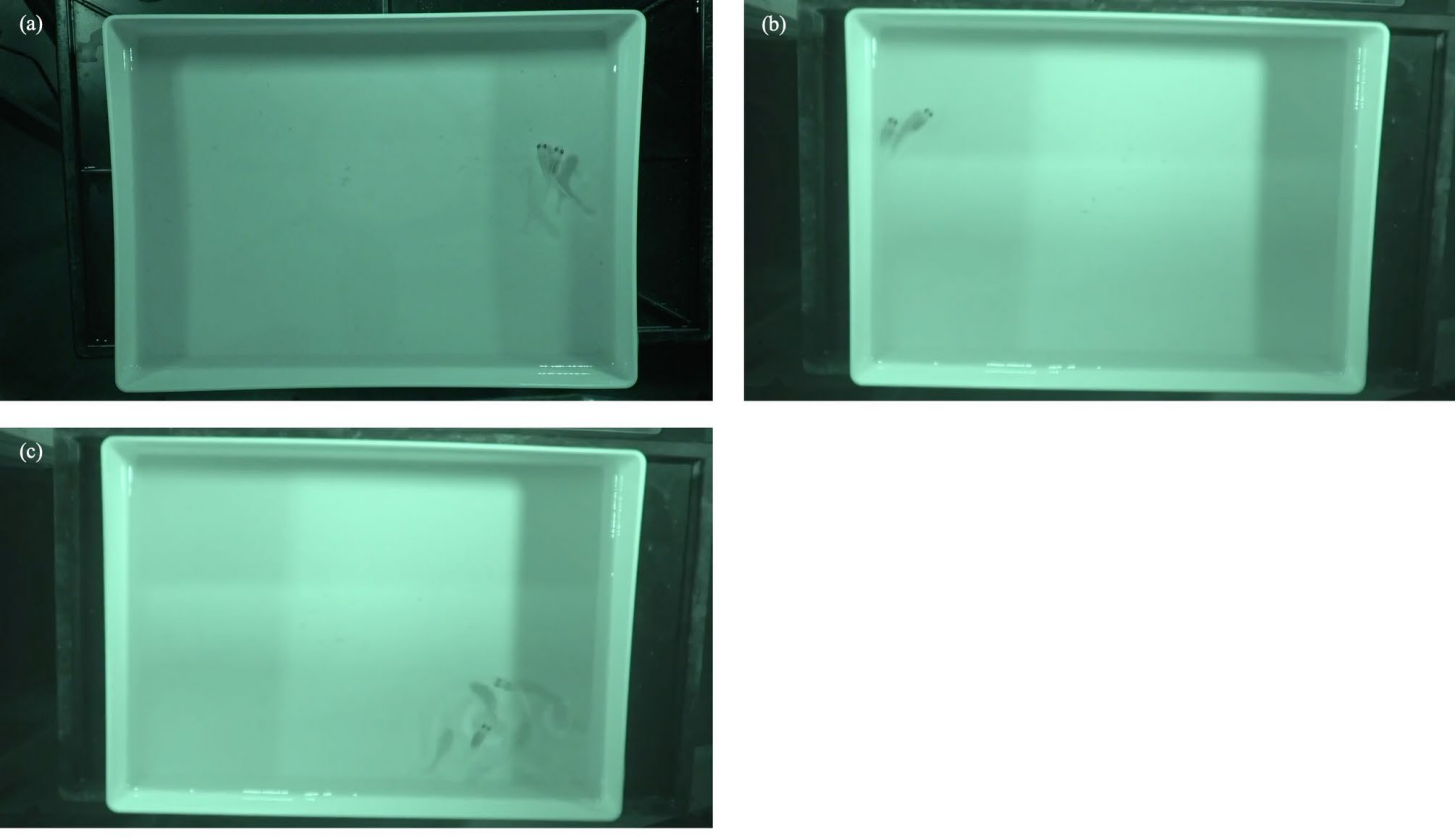
(**a**) Medaka spawning at night, captured using an infrared camera. The female was on the right, and the male was on the left. The male wrapped the female with his fins during egg and sperm release. See Supplementary Movie S1 for the video. (**b**) Following at night, captured by an infrared camera. The female was in the front right, and the male was behind her. The male was following the female. See Supplementary Movie S2 for the video. (**c**) Quick circling at night, captured by an infrared camera. The female was in the lower left, and the male was in front of her. The male was circling the female. See Supplementary Movie S2 for the video.

**Fig. 2:**
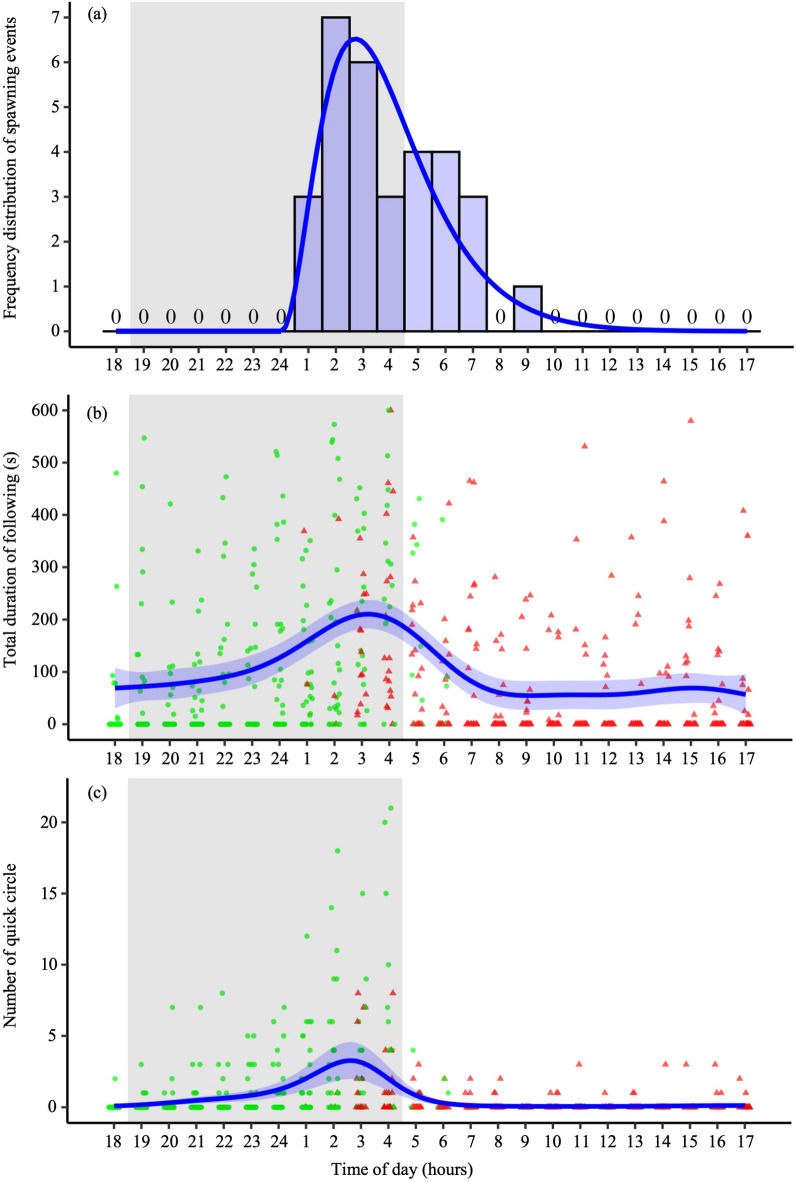
(**a**) Frequency distribution of spawning events observed each hour over 24 h in medaka, *Oryzias latipes* (*n* = 31). The peak of spawning events was determined using gamma distribution fitting. (**b**) and (**c**) Changes in courtship behaviours from 18:00 to 17:00 over 24 h. (**b**) The total following duration (sec / 10 min) and (**c**) the number of quick-circle behaviours (number/10 min). Each plot (green circle: pre-mating male; red triangle: post-mating male) signifies the observed values from the analysed videos. The regression curves were based on the generalised additive mixed models (GAMMs) using all data (*n* = 744 observations from 31 males), and the shading indicates the 95% confidence intervals.

Spawning occurred between 1:05 and 9:48 in all 31 pairs (Fig. 1a; Supplementary Movie S1). Peak spawning events were observed between 2:00–4:00 h (*n* = 13 of 31 spawning events; Fig. 2a; Supplementary Table S1); sunrise occurring at approximately 4:45 during the experimental period (Supplementary Table S2), only eight spawning events (26% of the total spawning events) were observed within 1 h before and after sunrise. Overall, 19 (61% of the total spawning events) and 12 (39%) spawning events occurred before and after sunrise, respectively.

### Temporal variations in courtship behaviour

A 24-h behavioural analysis was conducted for all 31 pairs in which spawning was confirmed to examine diurnal variations in courtship behaviour. “Following” was observed across all periods (74.31 ± 4.73 s per video segment, *n* = 744 observations from 31 males, mean ± S.E.; Fig. 1b; Supplementary Movie S2). Following a gradual increase from midnight, peaking between 2:00–5:00, and remained low after 9:00 (generalised additive mixed model [GAMM], deviance = -24.39, df = -5.47, *p* < 0.0001, *n* = 744 observations from 31 males; Fig. 2b). Although male following was less frequent after spawning, ca. ~55% of 31 post-mating males continued following females until 8:00, and ca. ~26% of 31 males did between 8:00–18:00 (Fig. 2b).

“Quick circle” was recorded 179 times during the observation period (0.70 ± 0.08 occurrences per video segment, *n* = 744 observations from 31 males; Fig. 1c; Supplementary Movie S3). The temporal trend of quick circle mirrored that of following behaviour, with an increase from midnight, peaking at 2:00–5:00, and remaining low after 6:00 (GAMM, deviance = -166.03, df = -8.27, *p* < 0.0001, *n* = 744 observations from 31 males; Fig. 2c). Unlike male-following behaviour, almost all post-mating males did not exhibit any quick-circle behaviours after 6:00 (Fig. 2c).

## Discussion

The timing of reproduction is a critical ecological trait in organisms; this directly affects the fitness of the individuals. Medaka spawning has traditionally been considered to occur within an hour before and after light onset^33–36^. However, these studies did not directly observe spawning behaviour under dark conditions; instead, they relied on estimations based on the developmental stage of eggs post-spawning. Consequently, the timing of the initiation of medaka spawning remains unclear. Recent field research by Kondo et al. (2025)^12^ provided initial evidence that challenged this view, as they observed females carrying fertilised eggs and higher courtship activity levels around midnight. Extending these findings, our study used infrared video cameras for nighttime observations under semi-outdoor conditions. It demonstrated that spawning behaviour begins as early as 1:00 and peaks at 2:00–4:00, even though sunrise occurs at approximately 4:45. Furthermore, courtship behaviours—following and quick circles began around midnight, peaking at 2:00–5:00. Therefore, spawning and courtship initiation in medaka occur much earlier than previously thought^33–36^.

In this study, following and quick circles, both courtship behaviours, gradually increased from midnight, reaching a peak before sunrise at 2:00–5:00. These findings suggest that medaka initiate courtship behaviour late at night. Previous laboratory studies on 24-h activity patterns in medaka have reported a gradual increase in activity, particularly from 4 to 5 h before light onset^38^. Although these studies only measured fluctuations in activity levels, leaving specific behaviours unexamined, the insights from this study on sunrise timing (approximately 4:45) and courtship initiation (around midnight) align with previously observed patterns of activity change. Consistent results were obtained between this study and Kondo et al. (2025)^12^, which has a limited methodology for indirect observation of spawning and observation duration (21:00–5:00), strengthening the conclusion that medaka reproductive activities begin in darkness. Our direct observations of spawning events complemented their field data, collectively confirming for the first time that increased activity before light onset is attributable to reproductive behaviour.

Even after mating, when no ovulated females were available, more than 50% and 25% of male medaka continued exhibiting persistent following behaviour until 8:00 and during 8:00–18:00, respectively. In contrast, the quick circle nearly disappeared after mating. These differences in courtship behaviours can be attributed to the following: Following behaviour first, the following is essential during the initial stage of courtship^31,45,46^, as it facilitates the search for potential mates. Second, because male medaka can reproduce multiple times a day^18,19^, they may attempt to search for mating partners even after a mating event. Third, the following behaviour may also function as mate guarding to ensure paternity^26^, preventing other males from accessing females carrying newly fertilised eggs. Some studies have suggested that mate-guarding can enhance male reproductive success through familiarisation with mating partners^25–27^, and there is evidence that female medaka tend to prefer familiar males^20^. Therefore, continued following behaviour could function as immediate mate-guarding and preparation for future reproduction. Conversely, a quick circle is the second step in courtship; males can recognise females’ ovulatory state^26,46^. Therefore, it is plausible that males can assess ovulatory states while following females and suppress quick circles if they determine that the female has already spawned. Thus, male medaka may employ context-dependent courtship behaviours that increase mating opportunities and reduce investment in courtship.

The timing of reproduction is a crucial ecological trait that has been evolutionarily shaped by multiple selective pressures, including predator avoidance, environmental adaptation, and mate synchronisation^1,9^. Nocturnal spawning is strongly associated with predator avoidance in adult parents and offspring because spawned eggs and newly hatched young individuals are the most vulnerable to predation during their life history^3^. Courtship behaviour, including visual, vocal, and chemical signals, often increases the risk of adult predation in many animals^48,49^. The benefits of nocturnal courtship in medaka may be related to predator avoidance strategies for both parents and eggs. In fish species where eggs are externally fertilised, such as medaka, spawning at dusk and night aligns with periods when the activity levels of planktonic predators targeting eggs and piscivorous predators targeting adult spawning are lower^50,51^. This ecological mechanism also applies to the medaka. Although research on the trophic level of medaka is limited, the main predators of adults include dragonfly larvae, birds, and piscivorous fish^52–54^, and the main egg predators observed in the field may be fish, such as bitterlings and dark chubs^12^. As the nocturnal activity levels of these predators are relatively low, medaka may engage in courtship and spawning events with reduced predation risk.

Medaka spawned without relying on visual cues^40^, and the significance of olfactory cues in the spawning process was demonstrated^46^. Additionally, the spawning capability under dark conditions was confirmed^39^. These findings suggest that males recognise ovulating females not only through visual cues but also through olfactory cues. The observed increase in courtship behaviour during the night aligns with previous findings. Medaka are presumed to begin courtship at midnight and initiate spawning shortly after female ovulation. However, further research is required to determine the exact timing of female ovulation. Fish communicate environmental and conspecific information using chemical cues^55–57^. Elucidating the male recognition mechanisms of spawning-ready females could enhance our understanding of medaka ecology and spawning behaviour.

Despite the novel insights provided by this study, several limitations must be acknowledged. First, although we propose that nocturnal spawning in medaka may be an adaptation to reduced predation risk, we did not directly measure predation pressure at different times of the day. Second, our observations were conducted in semi-natural environments rather than completely natural habitats, which may have influenced fish behaviour. Third, the study was conducted during a single season and at a single geographic location, potentially limiting our findings’ generalisability across different environmental conditions. Future research should address these limitations by using several approaches. Quantifying the predation pressure on medaka and their eggs at different times of the day would provide direct evidence for the predator avoidance hypothesis. Comparative studies of spawning timing among populations from different ecological niches could reveal how local adaptations shape reproductive timing. Additionally, investigating seasonal variations in spawning timing and how artificial light conditions affect reproductive behaviour would enhance our understanding of the interplay between endogenous rhythms and environmental cues (temperature, light, and predator cues) in regulating reproduction^15,16^.

Traditional studies on model organisms have predominantly been conducted in laboratory environments, where stringent environmental controls have contributed to understanding biological phenomena under simplified conditions^58^. However, because the natural habitats of organisms encompass far more complex factors than those present in laboratory settings, many ecological and behavioural aspects of model organisms remain insufficiently explored. Considering the importance of the medaka as a model organism, the findings of this study are significant beyond ecological discovery. The suggestion that reproductive behaviours observed in laboratory studies may capture only the latter part of the breeding period calls for re-evaluating the interpretation of laboratory findings. Future research directions include a comprehensive investigation of 24-h reproductive behaviour in medaka in their natural habitats and environmentally controlled laboratory settings to clarify the peak periods of courtship and other activities. These ecological and ethological approaches under (semi-) natural conditions are expected to deepen our understanding of biological phenomena’ fundamental mechanisms and functions as elucidated by observations conducted under controlled laboratory settings.

## Electronic supplementary material

Below is the link to the electronic supplementary material.

Supplementary Material 1

Supplementary Material 2

Supplementary Material 3

Supplementary Material 4

Supplementary Material 5

## Data Availability

Data are included as Supplementary Information.
