# Supplementary material for: Courtship and spawning behaviour of medaka in a semi-outdoor environment initiating at midnight
Source: Sci Rep. 2025 May 16;15:17057. doi: 10.1038/s41598-025-01037-8 (PMC12084311; doi:10.1038/s41598-025-01037-8)
Supplement: Supplementary file 3 — Supplementary Material 3 [file 41598_2025_1037_MOESM3_ESM.docx]

**Supplementary Materials**

**Table S1.** The experiment date, male and female body mass, and spawning time of the day.

| Male ID | Experiment date | Male body mass (g) | Female body mass (g) | Spawning time (JST) |
| --- | --- | --- | --- | --- |
| 1 | 2024/07/06 | 0.36 | 0.30 | 4:06 |
| 2 | 2024/07/07 | 0.38 | 0.28 | 1:16 |
| 3 | 2024/07/07 | 0.23 | 0.32 | 9:48 |
| 4 | 2024/07/07 | 0.32 | 0.32 | 7:32 |
| 5 | 2024/07/07 | 0.21 | 0.32 | 4:39 |
| 6 | 2024/07/07 | 0.29 | 0.26 | 1:05 |
| 7 | 2024/07/08 | 0.29 | 0.25 | 2:41 |
| 8 | 2024/07/08 | 0.33 | 0.40 | 6:57 |
| 9 | 2024/07/08 | 0.29 | 0.33 | 2:20 |
| 10 | 2024/07/08 | 0.32 | 0.37 | 3:50 |
| 11 | 2024/07/08 | 0.28 | 0.37 | 2:21 |
| 12 | 2024/07/09 | 0.24 | 0.32 | 5:24 |
| 13 | 2024/07/09 | 0.32 | 0.30 | 6:24 |
| 14 | 2024/07/09 | 0.28 | 0.25 | 3:00 |
| 15 | 2024/07/09 | 0.28 | 0.34 | 6:21 |
| 16 | 2024/07/10 | 0.24 | 0.30 | 4:10 |
| 17 | 2024/07/10 | 0.27 | 0.30 | 2:41 |
| 18 | 2024/07/10 | 0.27 | 0.28 | 2:53 |
| 19 | 2024/07/10 | 0.25 | 0.32 | 1:54 |
| 20 | 2024/07/10 | 0.29 | 0.34 | 5:14 |
| 21 | 2024/07/11 | 0.33 | 0.24 | 7:16 |
| 22 | 2024/07/11 | 0.27 | 0.34 | 5:24 |
| 23 | 2024/07/11 | 0.30 | 0.38 | 5:18 |
| 24 | 2024/07/11 | 0.33 | 0.39 | 7:00 |
| 25 | 2024/07/17 | 0.31 | 0.33 | 2:24 |
| 26 | 2024/07/17 | 0.27 | 0.33 | 6:16 |
| 27 | 2024/07/18 | 0.30 | 0.42 | 3:06 |
| 28 | 2024/07/18 | 0.40 | 0.33 | 3:19 |
| 29 | 2024/07/18 | 0.26 | 0.38 | 2:27 |
| 30 | 2024/07/19 | 0.32 | 0.35 | 3:13 |
| 31 | 2024/07/19 | 0.38 | 0.41 | 3:34 |

**Table S2.** Temperatures and sunrise and sunset time in Gifu, Japan, from July 6 to 12 and July 17 to 20, 2024.

| Date | Mean temperature (℃) | Max. temperature (℃) | Min. temperature (℃) | Sunrise time | Sunset time |
| --- | --- | --- | --- | --- | --- |
| July 6th | 30.5 | 34.4 | 27.2 | 4:44 | 19:11 |
| July 7th | 32.0 | 37.7 | 25.5 | 4:44 | 19:11 |
| July 8th | 31.9 | 36.4 | 28.4 | 4:45 | 19:11 |
| July 9th | 28.6 | 32.6 | 26.1 | 4:45 | 19:10 |
| July 10th | 27.6 | 31.0 | 25.4 | 4:46 | 19:10 |
| July 11th | 25.6 | 26.7 | 23.6 | 4:46 | 19:10 |
| July 12th | 24.5 | 28.2 | 22.4 | 4:47 | 19:09 |
| July 17th | 27.3 | 33.5 | 23.5 | 4:50 | 19:07 |
| July 18th | 29.9 | 35.8 | 24.5 | 4:51 | 19:07 |
| July 19th | 29.9 | 34.6 | 27.2 | 4:52 | 19:06 |
| July 20th | 29.2 | 35.4 | 27.1 | 4:53 | 19:06 |

**Table S3** Raw data for this study.

**Movie S1** A spawning event of medaka filmed at night using infrared camera.

**Movie S2** Following behaviour filmed at night using infrared camera.

**Movie S3** Quick circle behaviour filmed at night using infrared camera.
